# Communications of the Rhode Island Medical Society

**Published:** 1861-09

**Authors:** 


—The *Communications of the Rhode Island Medical Society*, for 1861, are made up of half a dozen papers from its members, the most interesting of which is a *Letter on Some Points of Military Surgery*, by the venerable Usher Parsons, M. D., of Providence. The use of the chlorate of potassa in phagædenic ulcerations, with cases illustrative, is detailed by W. Owen Brown, M. D. The salt is applied in solution, of varying strength, according to the severity of the ulceration—in one very threatening case of vulvitis, the salt in powder was freely sprinkled upon the diseased parts; and in ordinary stomatitis, Dr. Brown uses the chlorate, either in solution or intimately mixed with powdered sugar, and with the effect of arresting the ulcerative process very promptly.

"Weedon Cooke, Esq., Surgeon to the Royal Free Hospital, says: "It is invaluable in foul chronic ulcers of the legs; in tertiary sores not of an inflammatory character; in ulcers of the mouth and tongue, arising either from syphilis, or cancer, or *cancrum oris*, or necrosis of the jaws; and especially so in cleansing and deodorizing, and indeed, healing many parts of the body. I have employed it in all these lesions at the Cancer and the Royal Free Hospital, and am daily reminded of its estimable benefit wherever there is an absence of active inflammation."

